# Protective Effects of Fish Oil Against Brain Impairment in Rats with Chronic Ethanol-Induced Liver Damage Involving the NRF2 Pathway and Oxidative Stress

**DOI:** 10.3390/antiox14060704

**Published:** 2025-06-10

**Authors:** Qian Xiao, Yi-Hsiu Chen, Lu-Chi Fu, Herlin Ajeng Nurrahma, Jing-Huei Lai, Hitoshi Shirakawa, Suh-Ching Yang

**Affiliations:** 1 School of Nutrition and Health Sciences, Taipei Medical University, Taipei 11031, Taiwan; da07111002@tmu.edu.tw (Q.X.); da07113003@tmu.edu.tw (Y.-H.C.); ma07111003@tmu.edu.tw (L.-C.F.); da07111004@tmu.edu.tw (H.A.N.); 2 Core Laboratory of Neuroscience, Office of R&D, Taipei Medical University, Taipei 11031, Taiwan; m105095006@tmu.edu.tw; 3 Center for Neurotrauma and Neuroregeneration, Taipei Medical University, Taipei 11031, Taiwan; 4 Laboratory of Nutrition, Graduate School of Agricultural Science, Tohoku University, Sendai 980-8857, Japan; shirakah@m.tohoku.ac.jp; 5 Research Center of Geriatric Nutrition, College of Nutrition, Taipei Medical University, Taipei 11031, Taiwan; 6 Nutrition Research Center, Taipei Medical University Hospital, Taipei 11031, Taiwan; 7 School of Gerontology and Long-Term Care, College of Nursing, Taipei Medical University, Taipei 11031, Taiwan

**Keywords:** fish oil, alcoholic brain damage, oxidative stress, NRF2/KEAP1 pathway

## Abstract

Fish oil’s neuroprotective effects in ethanol-induced liver injury was investigated through the factor 2 (NRF2)/Kelch-like ECH-associated protein 1 (KEAP1) pathway. Male Wistar rats received a control liquid diet (C) or an ethanol diet (E), with 25% or 57% of fat replaced by fish oil (CF25, CF57, EF25, EF57) for 8 weeks. Compared to the C group, the E group exhibited brain damage, including impaired performance of Y maze and novel object recognition test, increased glial fibrillary acidic protein (GFAP)-positive astrocytes, and ionized calcium-binding adapter molecule 1 (Iba-1)-positive microglia. In the prefrontal cortex, glutathione (GSH) and phosphorylated (p)-NRF2 decreased, catalase activity increased, and *nqo1* mRNA declined; hippocampal NRF2 and *nqo1* were also downregulated. However, compared to the E group, the EF25 and EF57 groups exhibited restored spatial and memory functions, reduced GFAP and Iba-1 expressions, potentiated β-amyloid (Aβ) clearance, and escalated catalase activity. Furthermore, increases in p-NRF2 and elevated hippocampal *nqo1* mRNA expressions in the prefrontal cortex were observed in the EF25 and EF57 groups. In conclusion, fish oil ameliorated deficits in spatial and memory functions, and enhanced Aβ1-42 clearance in the prefrontal cortex and hippocampus of rats with chronic ethanol-induced liver damage by activating the NRF2/KEAP1 pathway.

## 1. Introduction

Approximately 17% of people aged 15 years and older worldwide have engaged in heavy episodic or binge drinking [1]. The latest World Health Organization (WHO) guidelines and a *Lancet* article clarified that there is no threshold amount of alcohol, and even the smallest volume has hazards [2,3]. Problematic episodes come from continuous heavy drinking [1]. Ethanol, the main toxic substance of alcohol beverages, is metabolized in two stages. First, ethanol is oxidized to acetaldehyde by three different axes: the microsomal ethanol oxidation system (MEOS) involving cytochrome P450 2E1 (CYP2E1), alcohol dehydrogenase (ADH), and catalase (CAT). Second, acetaldehyde is further oxidized to acetate by aldehyde dehydrogenase (ALDH) [4]. Reactive oxygen species (ROS) occur as byproducts of CYP2E1, ascribed to amassed oxidative stress in alcohol-related diseases [5].

Clinically, patients with alcohol use disorder frequently experience memory impairments, as well as behavioral and cognitive deficits [6,7,8]. Studies demonstrated that alcohol consumption is negatively associated with both gray matter and white matter volumes in the brain, and that binge drinking further exacerbates these negative effects on the brain’s structure [9]. Moreover, chronic alcoholic liver injury was also suggested to be correlated to memory deficits [10]. In the central nervous system (CNS), ethanol and acetate are primarily metabolized by neurons and astrocytes [11]. The sustained activation of these metabolic processes alters mitochondrial function and morphology, eventually leading to the dysregulation of oxidative stress [11]. The resulting oxidant overload, due to both alcoholic liver injury and local alcohol metabolism in the brain, induces a redox imbalance that further stimulates immune, apoptotic, and necrotic responses in neurons [12].

To counteract oxidative stress, cells activate nuclear factor erythroid 2-related factor 2 (NRF2) signaling, which is recognized as a crucial regulator in aging and various chronic diseases [13]. Under normal conditions, the Kelch-like ECH-associated protein 1 (KEAP1) complex binds to NRF2, maintaining its low levels through proteasomal degradation, but upon NRF2 recruitment to balance the redox status, protein kinase C (PKC) phosphorylates NRF2 at ser40 (p-NRF2(ser40)), leading to its detachment from KEAP1 [14]. p-NRF2 is translocated to nuclei and binds to the antioxidant response element (ARE), activating genes that code for enzymes involved in antioxidant defense and detoxification, such as NAD(P)H:quinone oxidoreductase 1 (NQO1) [15]. In addition, NRF2 plays a role in modulating inflammation by reducing an oxidative imbalance to prevent the activation of nuclear factor kappa–light-chain-enhancer of activated B cells (NF-κB) and inhibiting the degradation of NF-κB inducers, thus blocking NF-κB’s translocation to nuclei [16]. NRF2 signaling is therefore considered to be anti-inflammatory [16]. Chronic alcohol exposure was shown to disrupt the NRF2 system, contributing to neurodegenerative diseases, including Alzheimer’s disease (AD) and alcoholic-related brain disease (ARBD) due to β-amyloid (Aβ) aggregation in the brain [17,18,19].

Fish oil, composed primarily of eicosapentaenoic acid (EPA, 20:5) and docosahexaenoic acid (DHA, 22:6), is widely consumed as a supplement and has gained attention for its antioxidant and anti-inflammatory effects over recent decades [20]. Numerous studies proved that supplementing fish oil can alleviate metabolic diseases, such as metabolic dysfunction-associated steatotic liver disease (MASLD) [21], metabolic syndrome [22], and diabetes [23]. This protection is closely linked to the amelioration of oxidative stress [22]. NRF2 signaling was identified as a targeted pathway of polyunsaturated fatty acids (FAs; PUFAs), primarily through their electrophilic derivatives [24,25]. This provides a rationale for the beneficial effects of fish oil in neurodegenerative diseases such as Parkinson’s disease and AD [26,27]. Our previous study also demonstrated the potential benefits of fish oil in ARBD, focusing on insulin resistance by analyzing the insulin receptor substrate 1 (IRS-1)/glycogen synthase kinase-3β (GSK3β) pathway and ceramide concentration levels [28]. Therefore, we hypothesized that fish oil can reduce brain damage by mitigating oxidative stress through the activation of NRF2 signaling pathways in rats with chronic alcohol exposure. This study was designed to validate this hypothesis.

## 2. Materials and Methods

### 2.1. Animal Study Protocol

Male Wistar rats were purchased from BioLASCO Taiwan (Yilan, Taiwan). At the beginning age of 6 weeks, thirty-six rats were housed in plastic cages under a constant temperature at 23 ± 2 °C and a relative humidity of 55% ± 10%, in an individual animal facility with a 12 h light/dark cycle. This study was approved by the Institutional Animal Care and Use Committee of Taipei Medical University (approval no. LAC-2020-0297). During the adaption period, all rats were provided with LabDiet 5001 Rodent Diet (St. Louis, MO, USA) and water ad libitum. The ethanol induction model was established using Lieber–DeCarli’s model. The control liquid diet (C) consisted of olive oil, safflower oil, and corn oil, while the ethanol liquid diet (E) replaced maltodextrin with absolute alcohol (459396, Sigma-Aldrich, St. Louis, MO, USA) in an isocaloric formulation. To observe the effects of fish oil, both diets were modified by substituting olive oil with fish oil, and fish oil was included at concentrations of 25% and 57%, resulting in the following experimental groups: CF25 and CF57 for the control diet, and EF25 and EF57 for the ethanol diet. The final monounsaturated FA (MUFA)/PUFA ratios were 0.4, 0.7, and 1.6, with n–6/n–3 ratios of 21.9, 1.9, and 0.8, respectively. Behavioral patterns and blood samples collected from tail veins were examined before the experimental period for baseline values and at the end of the experiment. After 8 weeks, the rats were euthanized by the intraperitoneal injection of a combination of Rompun^®^ and Zoletil^®^50 (1:1, 1 mL/kg BW). Blood was collected from the ventral aorta, followed by collection of the liver and tissues from different anatomical regions of the brain for further analyses.

### 2.2. Blood Biochemical Examination

All blood samples were collected in heparin-containing vacutainers (BD Bioscience, Franklin Lakes, NJ, USA) and centrifuged at 3000 rpm and 4 °C for 15 min. Plasma was preserved for the examination of aspartate aminotransferase (AST) and alanine aminotransferase (ALT), which was performed by ADVIA^®^ Chemistry XPT (Siemens Healthineers, Forchheim, Germany).

### 2.3. Behavioral Tests

#### 2.3.1. Y Maze Spontaneous Alternation

The Y-shaped acrylic maze consisted of three arms (L 45 × W 10 × H 25 cm), each 120° apart. At the start of the experiment, a rat was placed in the center of the maze. Without interference, their instinctive visits to the three arms were recorded for 10 min. A clockwise or counterclockwise sequence of visits to each arm was defined as spontaneous alternation, scored as follows:$\frac{Number\;of\;spontaneous\;alternations}{Total\;number\;of\;arm\;entries-2}$.

#### 2.3.2. Novel Object Recognition (NOR) Test

The object recognition test was composed of a three-phase cycle of habituation, training, and testing. The main concept of the NOR test is the rats’ instinctive tendency to explore novel objects. A square (59 × 59 cm) flat-bottomed acrylic darkroom was used as the trial field. During habituation, the rats were allowed free access to the trail field for 10 min. After 24 h, the same procedure was conducted and recorded for the open-field test. In the training phase, two identical objects were introduced into the field for 10 min. The formal test was administered and recorded after 24 h, with one of the objects replaced by a novel one. The percentage of preference for the novel object was assessed using the following formula: $\frac{Time\;with\;novel\;object-time\;with\;original\;object\;}{Time\;with\;novel\;object+time\;with\;original\;object}$.

#### 2.3.3. Behavioral Analysis

All formal behavioral tests above were recorded with a fixed camera. Noldus EthoVision (Noldus Information Technology, Wageningen, The Netherlands) was used in this study to analyze behavioral patterns of the rats.

### 2.4. Antioxidant and Antioxidative Enzyme Activities

Liver tissues and the prefrontal cortex were homogenized in cold potassium phosphate lysate buffer (50 mM, with 1 mM EDTA, pH 7.5) at 1:10 (*w*/*v*). Glutathione (GSH), glutathione peroxidase (GPx), glutathione reductase (GRd), superoxide dismutase (SOD), and catalase (CAT) activities were measured using commercial kits (703002, 703102, 703202, 706002, and 707002, Cayman, Ann Arbor, MI, USA). All procedures were performed following the manufacturer’s instructions.

### 2.5. Histopathological Evaluation and Immunohistochemical (IHC) Staining

After dissection of the liver caudate lobe and brain, tissues were preserved in a 10% formaldehyde aqueous solution, followed by paraffin embedding. Tissue sections were stained with hematoxylin and eosin (H&E). All assessments were conducted by veterinary pathologists. Fatty changes and amounts of inflammatory cell foci were observed in the liver samples. Scores were calculated in a semiquantitative method. The scores for fatty changes were classified from 0 (none) to 4 (>67%). Inflammation scores were based on the number of inflammatory cell foci: fewer than 3 = 0, 4–10 foci = 1, 11–20 foci = 2, and so on, up to over 31 foci = 4 [29]. For the brain, morphologies of the cortical area (CA)1/CA2/CA3 were evaluated according to Wu and Wang’s method to access the viability of neuronal cells in the hippocampus [30]. GFAP and ionized calcium-binding adaptor molecule 1 (Iba-1) were stained by IHC to estimate the inflammatory status of microglial and astrocytes, respectively [28]. Images at ×200 magnification covering the CA1, CA2, and CA3 hippocampal subregions were used to quantify positively stained cells. The cell counts from all subregions were then aggregated for statistical analysis.

### 2.6. Western Blotting Analysis of the NRF2 Signaling Pathway

Liver, prefrontal cortical, and hippocampal tissues were lysed with radioimmunoprecipitation assay (RIPA) buffer containing protease and phosphatase inhibitors. The supernatants were preserved after centrifugation at 12,000 rpm and 4 °C for 30 min. Protein concentrations were quantified using the Bradford assay. Protein samples (50 μg) separated by sodium dodecyl sulfate polyacrylamide gel electrophoresis (SDS-PAGE) were transferred to polyvinylidene difluoride (PVDF) membranes. The procedures followed those of a previous study [31]. The antibodies of NRF2, p-NRF2(ser40), and KEAP1 for blotting are listed in Table A1. Briefly, the membranes were incubated with primary antibodies at room temperature for 1.5 h, followed by washing and incubation with respective secondary antibodies for another 1.5 h. After washing off the antibodies, the enhanced chemiluminescence (ECL) kit (JT96-K002, T-Pro Biotechnology, New Taipei City, Taiwan) was applied to visualize the bands. Images were captured using the UVP ChemiDoc It^®^ Imaging System (UVP LLC, Upland, CA, USA) and further analyzed with Image-Pro Plus (version 4.5, Media Cybernetics, Rockville, MD, USA). All values were normalized to internal control.

### 2.7. Reverse-Transcription Quantitative Polymerase Chain Reaction (RT-qPCR) Analysis

Liver and prefrontal cortical samples were extracted with the TRIzol reagent, while hippocampal RNA was extracted with an RNeasy Mini Kit (74104, Qiagen, Venlo, The Netherlands). Total RNA extracts were then reverse-transcribed into complementary (c)DNA using a synthesis kit (K1622, Thermo Fisher Scientific, Waltham, MA, USA). qPCRBIO SyGreen^®^ Mix (PB20.15, PCR Biosystems, London, UK) was applied for complementary (c)DNA amplification with the QuantStudio 1 Real-Time PCR System (Thermo Fisher Scientific). The primer sequences of nuclear factor, erythroid 2 like 2 (*nfe2l2*), *keap1*, heme oxygenase 1 (*hmox1)*, and *nqo1* are shown in Table A2.

### 2.8. Statistical Analysis

Values are presented as the mean ± standard deviation (SD), using GraphPad Prism 9 (GraphPad Software, Boston, MA, USA). Differences between the C and E groups were examined using the Student’s *t*-test. A one-way analysis of variance (ANOVA) was applied to compare differences among control diets (C, CF25, and CF57) and among ethanol diets (E, EF25, and EF57). A two-way ANOVA was used to analyze the effects of ethanol, fish oil, and their interaction.

## 3. Results

### 3.1. Ethanol Consumption, Body Weights (BWs), and Tissue Weights

The average daily liquid diet intake in the E, EF25, and EF57 groups was 77.5, 74.5, and 74.7 g, respectively, resulting in ethanol intake levels of around 3.7–3.9 g. A significantly lower BW in the 8th week was observed in the E group (Table 1). However, no differences in final BWs were found among the E, EF25, and EF57 groups (Table 1).

### 3.2. Hepatic Damage

**Figure 1:**
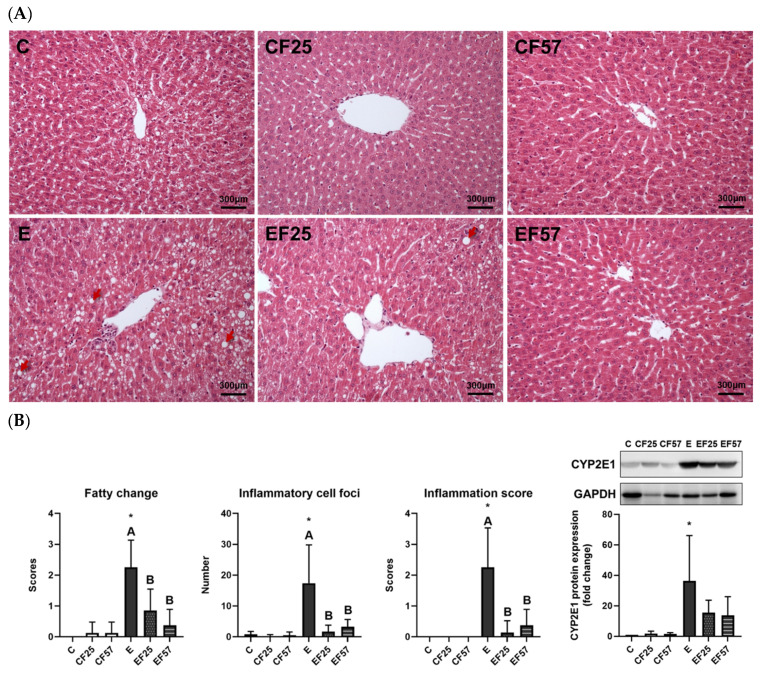
Effects of fish oil on hepatic damage markers in rats with alcohol-induced injury. (**A**) H&E staining (magnification ×200) (red arrows: lipid vacuoles). (**B**) Scores of fatty changes and inflammation (*n* = 5). (**C**) CYP2E1 protein expression (*n* = 6). * *p* < 0.05 compared to the C group by Student’s *t*-test. Superscript letters ^(A,B)^ indicate differences among three ethanol groups according to a one-way ANOVA with Tukey’s post hoc test (*p* < 0.05). C, control group; E, ethanol group; F25, 25% fish oil substituted for olive oil; F57, 57% fish oil substituted for olive oil; CYP2E1, cytochrome P450 2E1; GAPDH, glyceraldehyde 3-phosphate dehydrogenase.

Compared to the C group, higher plasma AST and AST activities were observed in the E group (Table 1). Hepatic impairments caused by chronic alcohol consumption were evident as escalated fatty changes and inflammatory foci in the E group (Figure 1, *p* < 0.001 compared to C). As the above evidence confirms, an alcohol-damaged liver disease model was established. EF25 demonstrated lower AST, with both the EF25 and EF57 groups exhibiting improved fatty changes and inflammatory foci by H&E observations and scores of fatty changes and inflammation (Table 1, Figure 1).

### 3.3. Hepatic Oxidative Hemostasis Shifts

**Figure 2:**
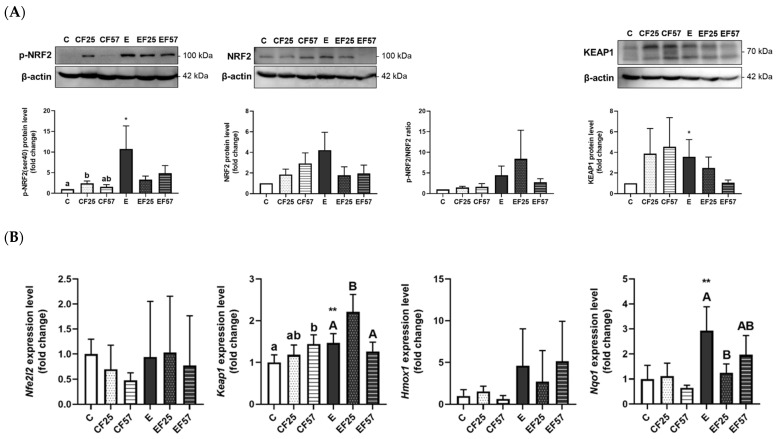
Effects of fish oil on the hepatic NRF2/KEAP1 signaling pathway in rats with chronic ethanol-induced liver damage (*n* = 6). (**A**) Representative images and quantitation of phosphorylated (p)-NRF2(ser40), NRF2, phosphorylated ratio, and KEAP1 protein expressions. (**B**) mRNA levels of *nfe2l2*, *keap1*, *hmox1*, and *nqo1*. Values are presented as the mean ± SD. * *p* < 0.05 compared to the C group by Student’s *t*-test. ** *p* < 0.01 compared to the C group according to Student’s *t*-test. Superscript letters (^a,b^) and (^A,B^), respectively, indicate differences among three control and ethanol groups according to a one-way ANOVA with Tukey’s post hoc test (*p* < 0.05). C, control group; E, ethanol group; F25, 25% fish oil substitution for olive oil; F57, 57% fish oil substitution for olive oil. NRF2, nuclear factor erythroid 2-related factor 2, KEAP1: kelch-like ECH-associated protein 1, nfe2l2, nuclear factor, erythroid 2 like 2; hmox1, heme oxygenase 1; nqo1, NAD(P)H:quinone oxidoreductase 1.

The GSH concentration and activities of antioxidant enzymes, including GPx, SOD, and CAT, significantly decreased in the E group. Among these enzymes, only CAT activity was restored in the EF25 and EF57 groups (Table 2). For the NRF2/KEAP1 pathway, protein expressions of p-NRF2 and KEAP-1 were significantly elevated in the E group, with increases in both *keap1* and *nqo1* mRNA levels (Figure 2). Compared to the E group, the EF25 group demonstrated significantly higher *keap1* and lower *nqo1* mRNA levels.

### 3.4. Behavioral and Histopathological Changes in the Brain

**Figure 3:**
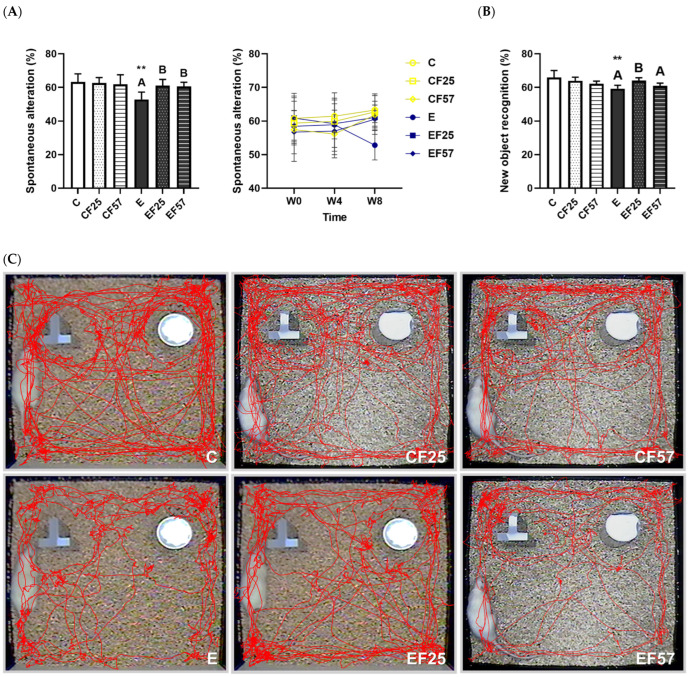
Effects of fish oil on behavioral changes in rats with chronic ethanol-induced liver damage (*n* = 6). (**A**) Spontaneous alterations in the Y maze. (**B**) Novel objective recognition (NOR) at the endpoint. (**C**) Representative images of rats’ route in the NOR test. Values are presented as the mean ± SD. ** *p* < 0.01 compared to the C group according to Student’s *t*-test. Superscript letters ^(A,B)^ indicate differences among three ethanol groups according to a one-way ANOVA with Tukey’s post hoc test (*p* < 0.05). C, control group; E, ethanol group; F25, 25% fish oil substitution for olive oil; F57, 57% fish oil substitution for olive oil.

The Y maze was used to assess the spatial working memory, with higher spontaneous alterations indicating better spatial memory [32]. The spontaneous alteration percentage was significantly lower in the E group (Figure 3A, *p* = 0.0028 C vs. E). The spontaneous alteration pattern was restored in both the EF25 and EF57 groups (Figure 3A). On the other hand, NOR served as an indicator of non-spatial learning and memory [33]. Rats in a normal condition tend to explore a novel object; therefore, an impaired recognition rate of novel objects was related to memory dysfunction [33]. Compared to the C group, exploration of the novel object was significantly reduced in the E group (*p* = 0.0056), while the EF25 group showed a significant increase in the recognition rate (Figure 3B).

**Figure 4:**
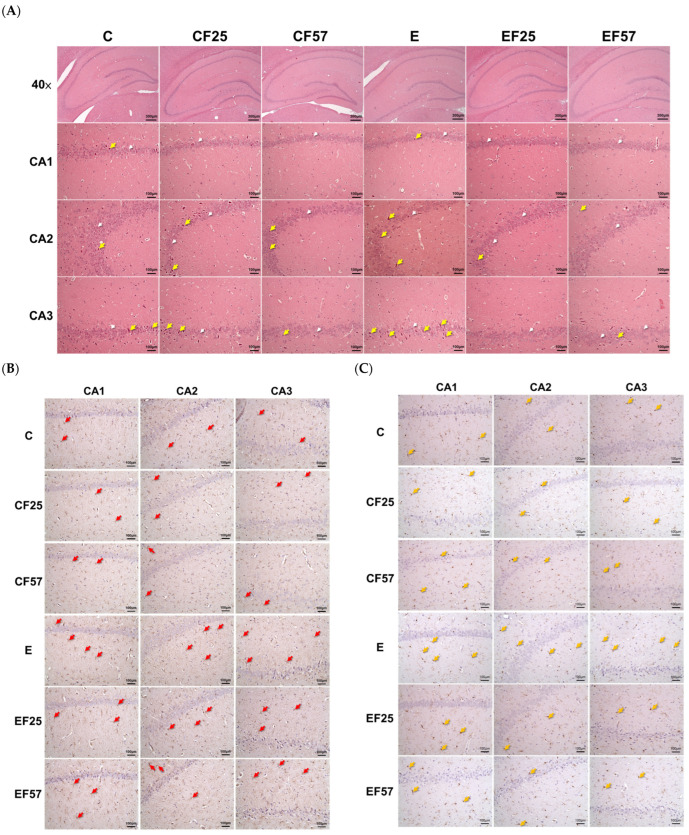

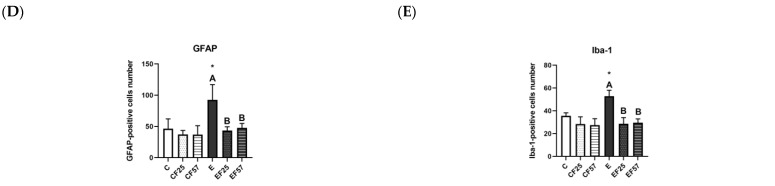
Effects of fish oil on hippocampus H&E staining and neuronal inflammation markers in rats with chronic ethanol-induced liver damage. (**A**) H&E staining of the hippocampus at ×40 magnification (white arrowhead: normal pyramidal cells, yellow arrow: nuclear condensed pyramidal cell). (**B**) Representative images of GFAP by IHC, at ×200 magnification (red arrow: GFAP-positive astrocytes). (**C**) Represented images of Iba-1 by IHC, at ×200 magnification (yellow arrows: Iba-1-positive microglia). (**D**) Cell counting analysis of GFAP-positive stained cells (*n* = 5). (**E**) Cell counting analysis of Iba-1-positive stained cells (*n* = 5). * *p* < 0.05 compared to the C group according to Student’s *t*-test. Superscript letters ^(A,B)^ indicate differences among three ethanol groups according to a one-way ANOVA with Tukey’s post hoc test (*p* < 0.05). E, ethanol group; F25, 25% fish oil substitution for olive oil; F57, 57% fish oil substitution for olive oil; CA, cortical area; GFAP, glial fibrillary acidic protein; Iba-1, ionized calcium-binding adapter molecule 1.

Regarding the histopathological examination of the hippocampus, the E group exhibited a greater degree of nuclear condensation in pyramidal cells within the CA1, CA2, and CA3 regions (Figure 4, C vs. E). GFAP-positive and Iba-1-positive staining appeared more frequently in the E group, indicating increased activation of astrocytes and microglia as injury markers (Figure 4B–E, C vs. E) [34]. Following fish oil administration, expressions of both GFAP and Iba-1 were reduced in the EF25 and EF57 groups (Figure 4B–E).

**Figure 5:**
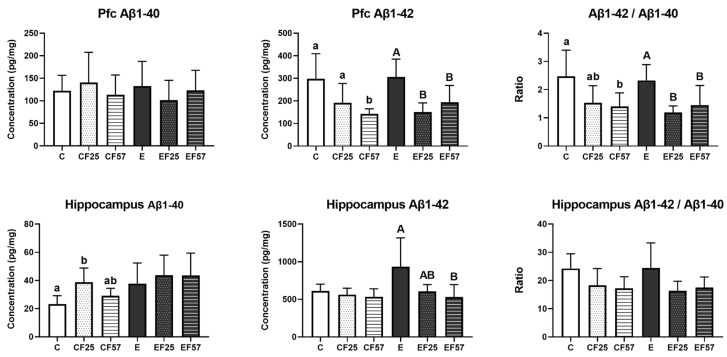
Effects of fish oil on prefrontal cortex and hippocampus β-amyloid (Aβ)1-40 and Aβ1-42 concentrations, and ratio in rats with chronic ethanol-induced liver damage (*n* = 6). Values are presented as the mean ± SD. Superscript letters ^(a,b)^ and ^(A,B)^, respectively, indicate differences among three control and ethanol groups according to a one-way ANOVA with Tukey’s post hoc test (*p* < 0.05). C, control group; E, ethanol group; F25, 25% fish oil substitution for olive oil; F57, 57% fish oil substitution for olive oil; Pfc, prefrontal cortex; Aβ, β-amyloid.

As to Aβ1-40 levels, the only notable change was higher hippocampal Aβ1-40 in the CF25 group compared to the C group. On the other hand, no significant differences in Aβ1-42 levels were observed in either the prefrontal cortex or hippocampus of the E group, although a trend toward significance was noted in the hippocampus when compared to the C group (*p* = 0.06, Figure 5). Both the EF25 and EF57 groups showed lower Aβ1-42 levels and ratios of Aβ1-42/Aβ1-40 compared to the E group in the prefrontal cortex, while in the hippocampus, only the EF57 group showed significantly lower Aβ1-42 levels (Figure 5, E vs. EF57).

### 3.5. Antioxidant Defense in the Prefrontal Cortex

The GSH concentration was significantly reduced in the E group compared to the C group (Table 3, *p* = 0.0270). However, no change was observed among the E, EF25, and EF 57 groups (Table 3). Regarding antioxidant enzymes, only CAT activity was markedly elevated in the E group (*p* = 0.0201, C vs. E), and the EF57 group also exhibited a higher level compared to the E group (Table 3).

### 3.6. The NRF2/KEAP1 Pathway in the Prefrontal Cortex and Hippocampus

**Figure 6:**
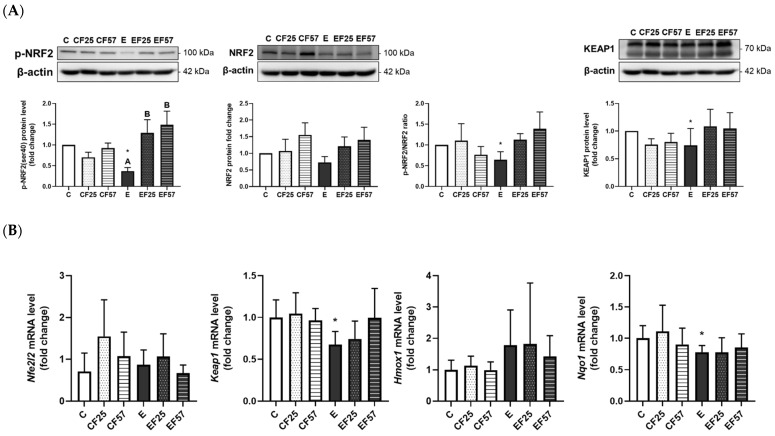
Effects of fish oil on prefrontal cortex NRF2/KEAP1 protein levels in rats with chronic ethanol-induced liver damage (*n* = 6). (**A**) Representative images and quantitation of phosphorylated (p)-NRF2(ser40), NRF2, phosphorylated ratio, and KEAP1 protein expressions. (**B**) mRNA levels of *nfe2l2*, *keap1*, *hmox1*, and *nqo1*. Values are presented as the mean ± SD. * *p* < 0.05 compared to the C group according to Student’s *t*-test. Superscript letters ^(A,B)^ indicate differences among three ethanol groups according to a one-way ANOVA with Tukey’s post hoc test (*p* < 0.05). C, control group; E, ethanol group; F25, 25% fish oil substitution for olive oil; F57, 57% fish oil substitution for olive oil.

In the prefrontal cortex, p-NRF2(Ser40) protein expression was significantly inhibited in the E group compared to the C group, whereas the EF25 and EF57 groups showed significantly higher p-NRF2(Ser40) protein expression than the E group (Figure 6A). This suppression was accompanied by a decrease in *nqo1* mRNA levels in the E group. Interestingly, *keap1* mRNA expression was also reduced in the E group (Figure 6B). However, no difference was found among the E, EF25, and EF 57 groups in terms of mRNA levels of *nqo1* or *keap1* (Figure 6B).

**Figure 7:**
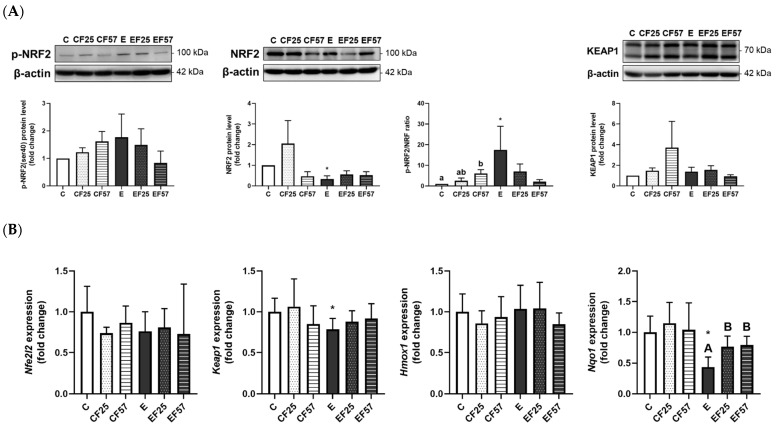
Effects of fish oil on hippocampal NRF2/KEAP1 protein levels in rats with chronic ethanol-induced liver damage (*n* = 6). (**A**) Representative images and quantitation of phosphorylated (p)-NRF2(ser40), NRF2, phosphorylated ratio, and KEAP1 protein expressions. (**B**) mRNA levels of *nfe2l2*, *keap1*, *hmox1*, and *nqo1*. Values are presented as the mean ± SD. * *p* < 0.05 compared to the C group according to Student’s *t*-test. Superscript letters ^(a,b)^ and ^(A,B)^, respectively, indicate differences among three control and ethanol groups according to a one-way ANOVA with Tukey’s post hoc test (*p* < 0.05). C, control group; E, ethanol group; F25, 25% fish oil substitution for olive oil; F57, 57% fish oil substitution for olive oil.

In the hippocampus, NRF2 protein expression was significantly inhibited, which contributed to a higher p-NRF2/NRF2 ratio; however, the *nqo1* mRNA level was significantly lower in the E group compared to the C group (Figure 7). Notably, *nqo1* mRNA levels were restored in the EF25 and EF57 groups (Figure 7B).

## 4. Discussion

### 4.1. Ethanol Administration Amount

The daily ethanol consumption was approximately 8.9–9.3 g/kg BW in the three ethanol-fed groups. This corresponds to roughly 90 g of ethanol for a 60 kg adult, equivalent to more than six standard drinks per day. According to the National Institute on Alcohol Abuse and Alcoholism (NIAAA), this meets the criteria for a heavy drinker [35]. The latest EASL-EASD-EASO Clinical Practice Guidelines suggest that this level of alcohol consumption in patients with steatotic liver diseases should be classified as alcoholic liver disease (ALD) [36].

### 4.2. Ethanol-Induced Hepatic Injury and Fish Oil

#### 4.2.1. Ethanol and Hepatic Injury

Rats fed ethanol-containing liquid diets without fish oil exhibited elevated AST and ALT activities, along with severe hepatic oil droplet accumulation, inflammatory cell infiltration, and CYP2E1 overexpression. Consistent with previous reports using the Lieber–DeCarli diet, alcohol-induced hepatic steatosis and inflammation were successfully established, resembling the early stage of ALD [29,37].

#### 4.2.2. Ethanol-Induced Hepatic Injury, Oxidative Stress, and the NRF2/KEAP1 Pathway

GSH levels and GPx, SOD, and CAT activities decreased after 8 weeks of ethanol intake (Table 2, C vs. E). However, p-NRF2 was found to be elevated in the E group, along with higher KEAP1 protein expression and mRNA levels (Figure 2, C vs. E). As the primary organ responsible for ethanol metabolism, when exposed to prolonged ethanol intake, the liver depletes alcohol dehydrogenase and over-activates CYP2E1 to oxidize ethanol. This leads to the increased production of reactive oxygen species (ROS) and other free radicals, further worsening the redox imbalance [38]. In response to overwhelming CYP2E1-derived oxidative stress, hepatocytes may enhance NRF2 signaling as an adaptive mechanism [39]. Not performing as expected, the antioxidant systems did not work in concert by upregulating NRF2. GSH and antioxidant enzymes are often found to show low abundances in patients with liver diseases and in chronic alcoholic rodent models [37,40,41]. This phenomenon may be attributed to the involvement of specific enzymes and antioxidants in ethanol metabolism and in the detoxification of its byproduct, hydrogen peroxide (H_2_O_2_) [42,43], despite activation of the NRF2 signaling pathway.

#### 4.2.3. Fish Oil and Ethanol-Induced Hepatic Injury

Fish oil ameliorated lipid accumulation and inflammatory foci and elevated CAT activity (Table 2, Figure 1, E vs. EF25, EF57). A UK Biobank study found that omega-3 PUFA supplementation significantly reduced the risk of ALD [44]. Omega-3 PUFAs were shown to mitigate alcohol-induced hepatic steatosis by activating free FA receptor 4 (FFA4) in Kupffer cells, leading to reduced lipid accumulation and inflammation [45]. In our previous study, we demonstrated that partial fish oil replacement may enhance FA oxidation by upregulating mRNA expressions of downstream FA-oxidative enzymes, such as medium-chain acyl-coenzyme A dehydrogenase (MCAD) and carnitine palmitoyl transferase (CPT)-1, ultimately attenuating ethanol-induced hepatic steatosis in rats subjected to chronic ethanol feeding [46].

On the other hand, the substitution of fish oil in the ethanol-liquid diet also increased hepatic CAT activity while decreasing the *nqo1* mRNA level (Table 2, Figure 2B; E vs. EF25, EF57). Siegel et al. confirmed that *nqo1* possesses SOD activity, both as a purified recombinant protein and in cellular systems expressing *nqo1* [47]. Therefore, the elevation of CAT activity observed with fish oil substitution might be related to enhanced ethanol clearance [48], thereby reducing the accumulation of oxidative adducts.

Additionally, *hmox1* and *nqo1* are phase II detoxifying enzymes regulated by upstream pathways and their expressions might not have been activated under the current experimental conditions [49]. The protective effect of fish oil may become more evident through the modulation of upstream regulators, such as inflammation-related signals like hypoxia-inducible factor (HIF)-1α [47].

### 4.3. Ethanol-Induced Brain Damage and Fish Oil

#### 4.3.1. Ethanol-Induced Behavioral Impairment and Brain Damage

Chronic alcohol exposure led to poorer performances in both the Y-maze and NOR tests, indicating impairments in spatial memory and recognition ability, respectively (Figure 3, C vs. E) [50]. Additionally, increased expressions of Iba-1 and GFAP in the E group suggested neuroinflammation or glial activation, which contributed to ethanol-induced brain damage (Figure 4A–E). Cognitive deficits associated with alcohol abuse are most frequently observed in visuospatial processing, memory, and executive function [51]. Spatial learning depends on the coordinated activity of the prefrontal cortex and hippocampus [52,53].

However, no significant Aβ accumulation was observed in the prefrontal cortex or hippocampus (Figure 5). Gong et al. reported that chronic ethanol consumption induces Aβ overproduction and oxidative stress in the mouse brain [54]. In their study, the Aβ level was measured using whole-brain samples, whereas in this study, a specific brain region was analyzed. This methodological difference may partly explain the lack of observable changes of Aβ overproduction induced by chronic ethanol intake in this study.

#### 4.3.2. Ethanol-Induced Brain Oxidative Stress and the NRF2/KEAP1 Pathway

When rats were subjected to chronic ethanol feeding, the GSH level was depleted, while CAT activity was elevated in the prefrontal cortex (Table 3, C vs. E). Moreover, protein expression levels of p-NRF2 and KEAP1 were suppressed, accompanied by a corresponding decrease in nqo1 mRNA levels in the prefrontal cortex (Figure 6, C vs. E). Taken together, although the NRF2 signaling pathway was inhibited, that did not suppress antioxidant enzyme activity; on the contrary, CAT activity increased. These findings suggest that CAT activity in the prefrontal cortex may be regulated through NRF2-independent pathways, possibly as a compensatory response to chronic ethanol-induced oxidative stress.

On the other hand, the hippocampus exhibited lower total NRF2 protein expression, which resulted in a higher ratio of p-NRF2 to total NRF2, along with a decreased mRNA level of *nqo1* under chronic ethanol intake (Figure 7, C vs. E). A previous study suggested that the prefrontal cortex is more vulnerable to alcohol exposure than the hippocampus, potentially due to DNA damage and impairments in one-carbon metabolism [55]. This regional difference in vulnerability may help explain the distinct molecular responses observed in this study, such as the more pronounced changes in NRF2 signaling and redox-related gene expressions in the prefrontal cortex. On the other hand, the hippocampus showed milder transcriptional changes, which may reflect a greater resilience to ethanol-induced oxidative stress. However, due to limited sample quantity, antioxidant enzyme activities in the hippocampus were not measured. Future studies with sufficient sample availability are warranted to assess enzyme activity levels in the hippocampus and further clarify its redox status under chronic ethanol exposure. As histological evidence of hippocampal injury was observed, a parallel analysis of the prefrontal cortex will be crucial for a more comprehensive understanding of region-specific vulnerability to ethanol-induced damage.

Fish oil improved spatial and short-term memory performances in rats under 8-week ethanol administration (Figure 3A,B, E vs. EF25, EF57). Fish oil also showed an ability to clear Aβ1-42 in both the prefrontal cortex and hippocampus (Figure 5, E vs. EF25, EF57). A systematic review suggested that omega-3 PUFA intake can enhance learning, memory, cognitive function, and cerebral blood flow [56]. Furthermore, omega-3 PUFA supplementation was reported to improve spatial and learning memory in both adult and adolescent male rats [57,58]. In addition, fish oil significantly attenuated chronic alcohol-induced aberrant dendritic morphological changes in medium-sized spiny neurons within both the core and shell regions of the nucleus accumbens [59]. Taken together, these findings support the protective role of fish oil in mitigating ethanol-related memory impairments.

In addition, from the perspective of Aβ-related brain damage, supplementation with EPA was shown to reduce Aβ concentrations, while omega-3 PUFAs might promote Aβ clearance through the glymphatic system [60,61]. As discussed above, Aβ accumulation did not appear after 8 weeks of ethanol administration. Nevertheless, fish oil enhanced Aβ scavenging, suggesting this as one of its protective properties in ARBD.

In the prefrontal cortex, p-NRF2 levels were significantly elevated, accompanied by increased CAT activity in rats fed an ethanol-containing diet substituted with fish oil (Figure 6, Table 3, E vs. EF25, EF57). Omega-3 PUFAs might activate the NRF2 pathway through multiple mechanisms. It was shown that omega-3 PUFAs activated the NRF2 signaling pathway, thereby upregulating antioxidant proteins such as heme oxygenase (HO)-1 in models of ischemic brain injury [62]. It was also indicated that modulation of dietary omega-3 PUFAs enhanced antioxidant protein expression in the hippocampus of mice through an NRF2/KEAP1-dependent mechanism [63]. Another key mechanism involves 4-hydroxy-2E-hexenal (4-HHE), a lipid peroxidation end-product of omega-3 PUFAs, which is known to be a potent NRF2 inducer [25]. In addition to the effects of reactive lipid species like 4-HHE, omega-3 PUFAs also integrate into cell membrane microdomains such as caveolae, which function as redox signaling hubs. Under basal conditions, a substantial fraction of NRF2 is localized in caveolae. Dietary Omega-3 PUFAs may incorporate into these membrane domains and modulate caveolin expression, thereby influencing membrane-associated signaling pathways that affect NRF2 activation [24]. Collectively, these findings suggest that fish oil may enhance antioxidant defense mechanisms through multiple pathways involving partial activation of the NRF2 signaling cascade.

Although total NRF2 protein expression and certain downstream antioxidant genes, such as *nqo1*, in the prefrontal cortex were not upregulated in the EF25 and EF57 groups (Figure 6, E vs. EF25, EF57), the observed dissociation between elevated p-NRF2(Ser40) protein levels and *nqo1* mRNA expression may be attributed to the presence of ethanol. Ethanol can cross the blood–brain barrier and undergoing local metabolism within the brain; however, the brain’s limited capacity for ethanol metabolism may influence cellular signaling and gene regulation pathways [64]. Notably, previous studies have reported that ethanol modulates histone deacetylase 2 (HDAC2) activity [65], which could subsequently interfere with NRF2-mediated transcriptional activation [66]. Additionally, nqo1 is a xenobiotic-metabolizing cytosolic enzyme whose expression may be regulated through multiple pathways [67]. Interestingly, the aryl hydrocarbon receptor (AhR), which responds to xenobiotics such as ethanol, may also modulate nqo1 expression independently of NRF2 [68]. This potential crosstalk could explain the lack of robust nqo1 induction despite increased levels of p-NRF2(Ser40) in this study (Figure 6, E vs. EF25, EF57). To more fully elucidate the functional consequences of NRF2 activation in this context, future studies should investigate additional downstream targets of NRF2, such as glutamate–cysteine ligase catalytic subunit (gclc), glutamate–cysteine ligase modifier subunit (gclm), and peroxiredoxin 1 (prdx1), which may exhibit greater responsiveness under chronic ethanol exposure and fish oil intervention. These molecular findings are consistent with behavioral improvements and histological protection observed in this study, further supporting the neuroprotective potential of fish oil supplementation under chronic ethanol exposure.

#### 4.3.3. Dosage of Fish Oil Substitution

In this study, the daily intake levels of fish oil in the EF25 (1.3 g/kg BW/rat) and EF57 (3.0 g/kg BW/rat) groups were equivalent to 12.5 and 28.8 g, respectively, in a human weighing 60 kg [69]. However, as no clear dose-dependent effect was observed in mitigating brain damage, we would like to emphasize the importance of the fatty acid composition rather than the absolute dosage. In the Lieber–DeCarli model, the fat source in the control liquid diet is modified and differs from standard rodent diets. One of the main rationales of our study design was to modulate the fatty acid profile. Specifically, the final MUFA to PUFA ratios were 0.4, 0.7, and 1.6, while the n-–6 to n–3 ratios were 21.9, 1.9, and 0.8, respectively. Therefore, we believe that adjusting the composition of dietary fatty acids with fish oil supplementation may be of greater importance than simply increasing the dosage in future clinical applications [46]. Further studies will be necessary to validate this concept.

#### 4.3.4. Research Limitations

Although this study provides important insights into the protective effects of fish oil supplementation under chronic ethanol exposure, several limitations remain that warrant further investigation. In the parameters of observation, limited brain tissue availability, antioxidant enzyme activities in the hippocampus and histopathological alterations in the prefrontal cortex were not analyzed. The mitochondrial function plays an important role both in alcoholic oxidative stress and redox in the CNS. Further, oxidative markers, such as malondialdehyde (MDA), 4-hydroxynonenal (4-HNE) or acrolein, which are involved in both alcohol damage and neurodegenerative diseases, could be considered for further investigation. Given the region-specific responses observed at the molecular level, it is essential for future studies to include both biochemical and histological assessments in these brain regions to establish clearer links between molecular changes and structural or functional outcomes. On the other hand, in consideration of clinical practice, we demonstrated the Lieber–DeCarli diet in male rats might restrict the perspective from the effects related to hormones and the accurately replicating the complex patterns of human alcohol consumption, particularly in terms of episodic intake and variable drinking behaviors. The exploration mentioned above might provide a more integrated picture of ARBD. Moreover, considering the well-recognized anti-inflammatory and antioxidant properties of fish oil [57], future research should also explore its effects through broader mechanistic lenses. These include the modulation of inflammatory signaling pathways and the roles of bioactive lipid mediators such as 4-HHE and ceramides, which may contribute to neuroprotection in ways beyond the NRF2 signaling pathway. A more holistic understanding of fish oil’s impacts on brain health may facilitate the development of nutritional strategies for preventing or attenuating alcohol-related neurotoxicity.

## 5. Conclusions

Fish oil ameliorated spatial and memory declines and potentiated Aβ1-42 clearance in the prefrontal cortex and hippocampus in rats with chronic ethanol-induced liver damage. The observed increase in p-NRF2(Ser40) and enhanced catalase activity in the prefrontal cortex may contribute to these protective effects. Additionally, the elevated *nqo1* mRNA expression in the hippocampus suggests that fish oil may help modulate redox balance in the brain under chronic ethanol exposure; however, the direct assessment of oxidative stress markers in the hippocampus is necessary to support this possibility.

## Figures and Tables

**Table 1:** Effects of fish oil on ethanol consumption, dietary intake and efficiency, body weight (BW), relative liver weight, and liver injury markers in rats with chronic ethanol-induced liver damage ^1^.

Group ^2^		-	F25	F57	Interaction (*p* Value) ^3^
					Ethanol × Fish Oil
Initial BW (g)	C	264.7 ± 13.0	271.2 ± 5.9	270.7 ± 11.8	0.5164
	E	271.4 ± 6.4	274.0 ± 9.9	267.7 ± 12.8	
Final BW (g)	C	449.9 ± 10.9	460.4 ± 8.0	442.3 ± 25.5	0.5559
	E	406.2 ± 21.2 *	404.2 ± 14.7	399.1 ± 11.8	
Relative liver weight (%)	C	3.06 ± 0.37	3.15 ± 0.19	3.15 ± 0.15	0.5370
	E	3.46 ± 0.41	3.85 ± 0.48	3.63 ± 0.30	
AST (U/L)	C	59.7 ± 5.8 ^a^	65.8 ± 5.8 ^a^	74.5 ± 3.3 ^b^	0.0058
	E	193.7 ± 61.3 *^A^	120.8 ± 38.9 ^B^	134.3 ± 19.6 ^A^	
ALT (U/L)	C	40.0 ± 12.1	49.5 ± 5.2	49.2 ± 7.8	0.6402
	E	96.0 ± 15.8 *	92.0 ± 32.8	93.7 ± 23.4	

^1^ Values are represented as the mean ± standard deviation (*n* = 6). An asterisk (*) indicates a significant difference compared to the C group (*p* < 0.05) according to Student’s *t*-test. Superscript letters ^(a,b)^ and ^(A,B)^, respectively, indicate differences among the three control and ethanol groups according to a one-way ANOVA with Tukey’s post hoc test (*p* < 0.05). ^2^ C, control group; E, ethanol group; F25, 25% fish oil substituted for olive oil; F57, 57% fish oil substituted for olive oil. ^3^ The interaction was evaluated by a two-way ANOVA with Tukey’s multiple-comparison test. BW, body weight; AST, aspartate aminotransferase; ALT, alanine aminotransferase.

**Table 2:** Effects of fish oil on hepatic glutathione (GSH) concentration and antioxidant enzymes in rats with chronic ethanol-induced liver damage ^1^.

Group ^2^		-	F25	F57	Interaction (*p* Value) ^3^
					Ethanol × Fish Oil
GSH (µM/mg)	C	35.9 ± 18.11	23.34 ± 8.4	21.19 ± 4.63	0.0247
	E	10.20 ± 2.66 *	12.31 ± 4.07	13.93 ± 6.35	
GPx (nmol/min/mg)	C	0.225 ± 0.055	0.241 ± 0.083	0.174 ± 0.054	0.3770
	E	0.147 ± 0.035 *	0.175 ± 0.057	0.149 ± 0.027	
GRd (nmol/min/mg)	C	12.336 ± 2.393	12.2 ± 1.793	12.85 ± 1.82	0.2203
	E	9.429 ± 2.892	12.996 ± 4.46	12.188 ± 3.261	
SOD (U/mg)	C	13.27 ± 2.97	14.98 ± 4.11	13.68 ± 4.23	0.7607
	E	8.79 ± 2.27 *	12.15 ± 3.95	10.85 ± 3.12	
CAT (nmol/min/mg)	C	4705.4 ± 1886.4	4638.2 ± 1136.7	5344.4 ± 864.6	0.4142
	E	2522.3 ± 724.2 *^A^	3062.4 ± 458.5 ^B^	4222.9 ± 921.0 ^B^	

^1^ Values are presented as the mean ± standard deviation (*n* = 6). An asterisk (*) indicates a significant difference compared to the C group (*p* < 0.05) according to Student’s *t*-test. Superscript letters ^(A,B)^ indicate differences among three ethanol groups according to a one-way ANOVA with Tukey’s post hoc test (*p* < 0.05). ^2^ C, control liquid diet group; E, ethanol liquid diet group; F25, fish oil substituted for 25% olive oil in dietary oil; F57, fish oil substituted for 57% olive oil in dietary oil. ^3^ The interaction was evaluated by a two-way ANOVA with Tukey’s multiple-comparison test. GSH, glutathione; GPx, glutathione peroxidase; GRd, glutathione reductase; SOD, superoxide dismutase; CAT, catalase.

**Table 3:** Effects of fish oil on prefrontal cortex glutathione (GSH) concentration and antioxidant enzymes in rats following chronic alcohol feeding ^1^.

Group ^2^		-	F25	F57	Interaction (*p* Value) ^3^
					Ethanol × Fish Oil
GSH (µM/mg)	C	20.11 ± 4.03	13.81 ± 8.11	12.05 ± 5.25	0.4606
	E	12.31 ± 6.18 *	11.18 ± 4.37	15.27 ± 5.62	
GPx (nmol/min/mg)	C	283.89 ± 39.03	230.21 ± 52.66	240.74 ± 47.79	0.0798
	E	279.35 ± 91.85	274.37 ± 78.98	306.15 ± 87.59	
SOD (U/mg)	C	7.96 ± 1.32	6.71 ± 1.34	5.91 ± 1.38	0.7309
	E	7.48 ± 2.89	7.03 ± 0.6	6.4 ± 1.04	
CAT (nmol/min/mg)	C	6.95 ± 3.3	9.33 ± 3.25	9.64 ± 2.03	0.2093
	E	11.60 ± 2.48 *^A^	13.19 ± 2.89 ^AB^	17.79 ± 4.09 ^B^	

^1^ Values are presented as the mean ± standard deviation (*n* = 6). An asterisk (*) indicates a significant difference compared to the C group (*p* < 0.05) according to Student’s *t*-test. Superscript letters ^(A,B)^ indicate differences among three ethanol groups according to a one-way ANOVA with Tukey’s post hoc test (*p* < 0.05). ^2^ C, control liquid diet group; E, ethanol liquid diet group; F25, fish oil substituted for 25% olive oil in dietary oil; F57, fish oil substituted for 57% olive oil in dietary oil. ^3^ The interaction was evaluated by a two-way ANOVA with Tukey’s multiple-comparison test.

**Figure A1:**
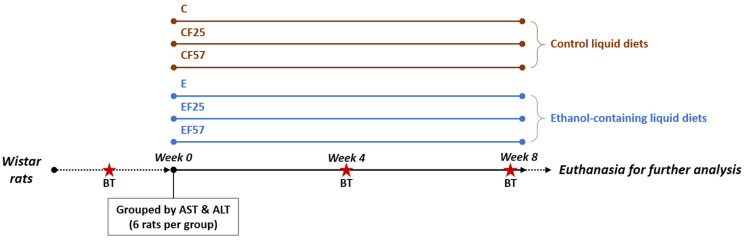
Schematic diagram for experimental design. After one week of adaption, all rats were distributed into 6 groups (*n* = 6) with no AST and ALT differences among groups. Behavior tests were conducted before grouping, followed by the 4th week and the endpoint of the experiment. C, control group; E, ethanol group; F25, 25% fish oil substituted for olive oil; F57, 57% fish oil substituted for olive oil; BT, behavioral tests; AST, aspartate aminotransferase; ALT, alanine aminotransferase.

## Data Availability

The data that support the findings of this study are available from the corresponding author upon reasonable request.

## Abbreviations

The following abbreviations are used in this manuscript:

4-HHE	4-hydroxy-2E-hexenal
AD	Alzheimer’s disease
ADH	alcohol dehydrogenase
ALD	alcoholic liver disease
ALDH	aldehyde dehydrogenase
ALT	alanine aminotransferase
ANOVA	analysis of variance
ARBD	alcoholic-related brain disease
ARE	antioxidant response element
AST	aspartate aminotransferase
Aβ	β-amyloid
BW	body weight
CAT	catalase
CNS	central nervous system
CPT	carnitine palmitoyl transferase
CYP2E1	cytochrome P450 2E1
DHA	docosahexaenoic acid
EPA	eicosapentaenoic acid
GAPDH	glyceraldehyde 3-phosphate dehydrogenase
GFAP	glial fibrillary acidic protein
GPx	glutathione peroxidase
GRd	glutathione reductase
GSH	glutathione
GSK3β	glycogen synthase kinase-3β
HIF	hypoxia-inducible factor
hmox1	heme oxygenase 1
HO	heme oxygenase
Iba-1	ionized calcium-binding adapter molecule 1
IHC	histopathological evaluation and immunohistochemical
IRS-1	insulin receptor substrate 1
KEAP1	kelch-like ECH-associated protein 1
MASLD	metabolic dysfunction-associated steatotic liver disease
MCAD	medium-chain acyl-coenzyme A dehydrogenase
MEOS	microsomal ethanol oxidation system
MUFA	monounsaturated fatty acid
nfe2l2	nuclear factor, erythroid 2 like 2
NF-κB	nuclear factor kappa-light-chain-enhancer of activated B cells
NIAAA	National Institute on Alcohol Abuse and Alcoholism
NOR	novel object-recognition
NQO1	NAD(P)H:quinone oxidoreductase 1
NRF2	nuclear factor erythroid 2-related factor 2
PKC	protein kinase C
PUFAs	polyunsaturated fatty acids
PVDF	polyvinylidene difluoride
RIPA	radioimmunoprecipitation assay
ROS	reactive oxygen species
SDS-PAGE	sodium dodecyl sulfate polyacrylamide gel electrophoresis
SOD	superoxide dismutase
WHO	World Health Organization

## Appendix A

**Table A1:** Antibodies used for Western blotting.

	Antibody	Type	Product No.	Source	Dilution
Primary antibody	CYP2E1	polyclonal	AB1252	Millipore	1:10,000
	p-NRF2(ser40)	polyclonal	bs-2013R	Bioss Antibodies	1:1000
	NRF2	polyclonal	16396-1-AP	Proteintech Group	1:1000
	KEAP1	polyclonal	10503-2-AP	Proteintech Group	1:2000
Internal controls	β-actin	monoclonal	sc-47778	Santa Cruz Biotechnology	1:1000
	GAPDH	monoclonal	MAB374	Millipore	1:600
Secondary antibody	anti-mouse IgG	HRP conjugated	#405306	BioLegend	1:5000
	anti-rabbit IgG	HRP conjugated	#406401	BioLegend	1:5000

**Table A2:** Primers used for RT-qPCR.

	Forward 5′→3′	Reverse 3′→5′	GenBank No.
NFE2L2	CTCTCTGGAGACGGCCATGACT	CTGGGCTGGGGACAGTGGTAGT	NM_001399173.1
KEAP1	TGGGTCAAATACGACTGCCC	GCAGGATCTCGCACTTTTGC	NM_057152.2
HMOX1	GAGCGAAACAAGCAGAACCC	ACCTCGTGGAGACGCTTTAC	NM_012580.2
NQO1	TCCGAAGCATTTCAGGGTCG	TCTGCGTGGGCCAATACAAT	NM_017000.3
GAPDH	AGTGCCAGCCTCGTCTCATA	GATGGTGATGGGTTTCCCGT	NM_017008.4
